# Genome Diversity and Signatures of Selection for Production and Performance Traits in Dromedary Camels

**DOI:** 10.3389/fgene.2019.00893

**Published:** 2019-09-19

**Authors:** Hussain Bahbahani, Hassan H. Musa, David Wragg, Eltahir S. Shuiep, Faisal Almathen, Olivier Hanotte

**Affiliations:** ^1^Department of Biological Sciences, Faculty of Science, Kuwait University, Kuwait City, Kuwait; ^2^Department of Medical Microbiology, Faculty of Medical Laboratory Sciences, University of Khartoum, Khartoum North, Sudan; ^3^Centre for Tropical Livestock Genetics and Health, The Roslin Institute, Edinburgh, United Kingdom; ^4^Department of Animal Production, Faculty of Agricultural and Environmental Sciences, University of Gadarif, Gadarif State, Sudan; ^5^Department of Public Health, College of Veterinary Medicine, King Faisal University, Al-Hasa, Saudi Arabia; ^6^LiveGene, International Livestock Research Institute (ILRI), Addis Ababa, Ethiopia

**Keywords:** genotype-by-sequence, positive selection, milk production, racing camels, Arabian Peninsula, Sudan

## Abstract

Dromedary camels (*Camelus dromedarius*) are single-humped animals found throughout the deserts of Africa, the Arabian Peninsula, and the southwest of Asia. This well-adapted species is mainly used for milk and meat production, although some specific types exhibit superior running performance and are used in racing competitions. However, neither performance nor production camels are bred under intensive genomic selection programs with specific aims to improve these traits. In this study, the full genome sequence data of six camels from the Arabian Peninsula and the genotyping-by-sequencing data of 44 camels (29 packing and 15 racing) from Sudan were analyzed to assess their genome diversities, relationships, and candidate signatures of positive selection. Genome ADMIXTURE and principle component analyses indicate clear geographic separation between the Sudanese and the Arabian Peninsula camels, but with no population-specific genetic distinction within populations. Camel samples from the Arabian Peninsula show higher mean heterozygosity (0.560 ± 0.003) than those from Sudan (0.347 ± 0.003). Analyses of signatures of selection, using pooled heterozygosity (*Hp*) approach, in the Sudanese camels revealed 176, 189, and 308 candidate regions under positive selection in the combined and packing and racing camel populations, respectively. These regions host genes that might be associated with adaptation to arid environment, dairy traits, energy homeostasis, and chondrogenesis. Eight regions show high genetic differentiation, based on *Fst* analysis, between the Sudanese packing and racing camel types. Genes associated with chondrogenesis, energy balance, and urinary system development were found within these regions. Our results advocate for further detailed investigation of the genome of the dromedary camel to identify and characterize genes and variants associated with their valuable phenotypic traits. The results of which may support the development of breeding programs to improve the production and performance traits of this unique domesticated species.

## Introduction

The *Camelidae* family is divided into two tribes, the New World camel (Lamini) and the Old World camel (Camelini). Within the Camelini, there are two domesticated species, the two-humped Bactrian camel (*Camelus bactrianus*) and the single-humped dromedary camel (*Camelus dromedarius*), in addition to the critically endangered two-humped wild Bactrian camel (*Camelus ferus*) found in northern China and southern Mongolia (Wilson, 1998).

Out of approximately 35 million camel heads worldwide (FAO, 2019), the majority (95%) are of the dromedary type (Hashim et al., 2015). Unlike the Bactrian camels, which are distributed throughout central and eastern Asia, dromedary camels mainly populate the desert and semi-desert areas across Africa, the Arabian Peninsula, and southwest of Asia (Wilson, 1998). They are highly adapted to the harsh desert environment, which is characterized by high temperatures and scarcity of food and water. Dromedary camels are tolerant to temperatures in excess of 40°C and can survive for up to 20 to 35 days without water, losing up to 25% of their body weight (Schmidt-Nielsen, 1959; Musa et al., 2006).

Archaeological evidence indicates dromedary camels to have been domesticated in the southeast of the Arabian Peninsula in the late second millennium BC (Magee, 2015), following which they spread to the northern part of the Arabian Peninsula and Africa *via* ancient trading routes. Being highly adapted to the desert environment, dromedary camels were historically the preferred means of transporting people and goods along routes such as the “incense road”—the trans-Arabian trading route used to transport valuable spices and perfumes from southern to northern areas of the Arabian Peninsula. The connection between the Arabian Peninsula and Africa was first established in East of Africa, through the Islands of Socotra, in association with sea-borne incense trading during the 1^st^ millennium BC (Epstein and Mason, 1971). In parallel, luxury goods, such as ivory, wools, and skins, were also transported from Africa to the Arabian Peninsula through Aden (Andree-Salvini, 2010). By the 7^th^ century, dromedary camels were widespread throughout the African Sahara employed in the transport of goods (Gauthier-Pilters and Anne, 1983). The spread of Islam to Africa at that time also contributed to the spread of dromedary between Africa and the Arabian Peninsula, as they were used to transport people to Makkah in the Arabian Peninsula during the annual pilgrimage “*hajj*” (Wilson, 1998).

The majority of dromedary, around 80%, are found in Africa. Sudan accommodates around 4.8 million heads, second in size to Somalia (Ali et al., 2019), representing around 22% of the animal biomass in the country (Eisa and Mustafa, 2011). The history of dromedary camel in Sudan can be traced to 15–25 years BC following human migrations from Egypt (Eisa and Mustafa, 2011; Hashim et al., 2015). Camels inhabit an area in Sudan characterized by arid conditions and erratic periods of rainfall. The area is flanked by the Butana plains and Red Sea hills in the East, and the Darfur and Kordofan States in the West (Eisa and Mustafa, 2011). Here, camels are classified as either “packing” or “racing” with the former type being used for milk and meat production, while the latter is typically used in racing competitions. Packing camel populations, such as the Arabi, Kenanai, Lahawi, and Rashaydi, constitute around 80% of a nomad’s herd and are characterized by their heavy weight and for having a well-developed hump (Hashim et al., 2015). Milk production in these camels is in the range of 820–2,400 liters per lactation period, which typically lasts for 12–18 months. The level of productivity depends mainly on the season and the farming management system (Faye et al., 2011). Other camel populations in Sudan, such as the Anafi and Bishari, which are famous for their racing performance, are lighter than the pack camels and are generally found in the northeast of Sudan and the River Nile State (Wardeh, 2004; Hashim et al., 2015). Populations of each of these camel types in Sudan are named after the ethnic groups that rear them.

Dromedaries in the Arabian Peninsula follow a similar classification system to that in Africa (Wardeh, 2004) but are further classified according to their coat color. Black dromedary camels are called Majaheem, whereas white and brown camels are called Wodh and Shual, respectively. A further type also characterized by a brown coat, but darker than Shual on the hump and tail, is called Sofor (Al-Swailem et al., 2007; Almathen et al., 2018). Each of these types are considered to be packing animals used for meat and milk production, with the Majaheem type being the most popular (Wardeh, 2004). Other popular types include the Omani originating from Oman, and the Hura from Saudi Arabia, which are both considered high-performance racing camels (Wardeh, 2004). Despite the different classifications and livestock functions, no selection programs aimed at improving the different types are in place.

Until recently, genetic analyses of dromedary camel have been largely restricted to studies involving autosomal microsatellite markers (Mburu et al., 2003; Almathen et al., 2016), partial mitochondrial DNA (mtDNA) sequences (Babar et al., 2015; Almathen et al., 2016) and candidate gene sequencing (Pauciullo et al., 2013; Shuiep et al., 2013; Almathen et al., 2018). These tools have mainly been employed to assess the genetic diversity and structure of dromedary camel populations from different geographical locations, e.g., Kenya (Mburu et al., 2003), the Arabian Peninsula (Almathen et al., 2016), and Pakistan (Babar et al., 2015). Efforts to link genotypes to phenotypes have also been undertaken—for example, Almathen et al. (2018) linked an arginine to cysteine missense mutation at protein position 301 in the melanocortin 1 receptor (*MC1R*) gene to white coat color. In the same study, a 1-bp deletion (23del T/T) and a SNP (25G/A) in exon 2 of the agouti signaling protein (*ASIP*) gene were linked to the black and dark-brown coat color phenotypes of Saudi Arabian dromedary. Recently, Khalkhali-Evrigh et al. (2018) analyzed the full genomes of two Iranian dromedary camels and reported non-synonymous variants in genes related to adaptation to the desert environment.

Signatures of selection analyses in African indigenous livestock have been conducted in different breeds or populations for a number of species, including, for instance, cattle (Bahbahani et al., 2015; Bahbahani et al., 2017; Kim et al., 2017a; Bahbahani et al., 2018a; Bahbahani et al., 2018b), goat (Kim et al., 2016; Onzima et al., 2018), and sheep (Kim et al., 2016), but to date, nothing has been reported for dromedary. Reference genomes of dromedary camels from the Arabian Peninsula and North Africa were published in 2014 and 2015, respectively (Wu et al., 2014; Fitak et al., 2015); however, neither is very contiguous, being assembled into 32,573 and 35,752 scaffolds, respectively. These assemblies are a fundamental first-step toward facilitating genome-wide analyses for signatures of selection. For example, Wu et al. (2014) analysis of the Arabian Peninsula dromedary genome revealed accelerated evolution and positive selection in genes related to fat metabolism, heat stress response, and salt metabolism.

Although the costs of whole-genome sequencing (WGS) have declined significantly in recent years, it remains an expensive endeavour to sequence large numbers of individuals, with large and complex genomes, to enable genome-wide studies. For some livestock species such as cattle and sheep, high-density genotyping microarrays are available enabling hundreds of thousands of genomic positions to be genotyped at a fraction of the cost of WGS (Rincon et al., 2011; Kijas et al., 2014). Where these tools are unavailable, typically in the case of non-model species with poor genomic resources, an alternative approach is to employ a genotyping-by-sequencing (GBS) strategy. GBS is a high-throughput multiplex sequencing system based on constructing reduced representative libraries for subsequent sequencing (Elshire et al., 2011). As with genotype microarrays, GBS can be performed at a fraction of the cost of WGS and does not suffer the ascertainment bias associated with array-based genotyping (Poland and Rife, 2012). The main disadvantage of this approach, however, is that the distribution of coverage throughout the genome is generally not uniform, and as a result, there is typically extensive between-sample variation in the genomic regions sequenced (Beissinger et al., 2013). GBS has been widely used in plant breeding for genome-wide association analysis, genomic diversity studies, and genomic selection (reviewed in He et al. (2014)). In addition, Wang et al. (2018) employed GBS to analyze the genomes of Chinese Landrace and Yorkshire pigs, identifying candidate signatures of selection in genes related to fatty acid biosynthesis, animal growth and development, and immune responses.

The availability of reference genome assemblies coupled with GBS enables us to explore in greater detail than ever before the genomes of different dromedary populations and to seek to identify genetic associations with different phenotypic traits. The aim of this study is to exploit these resources to assess the genomic diversity and relationship of dromedary camel populations from Sudan and the Arabian Peninsula and to undertake signatures of selection analyses to identify genes that might be associated with environmental adaptation, milk production, and racing performance in the Sudanese camels.

## Materials and Methods

### Animal Resources

Five Sudanese indigenous camel populations were sampled for this study. These included three packing camel populations: Arabi from western Sudan (n = 9), Kenani from central Sudan (n = 10), and Lahawi from eastern Sudan (n = 10) and two racing camel populations from eastern Sudan: Bishari (n = 7) and Anafi (n = 8) (**Table 1** and **Supplementary Figure S1**). Six camel samples from the Arabian Peninsula were also included in this study: three Majaheem, two Omani, and one Sofor. Two Majaheem samples were collected from the Conservation and Genetic Improvement Centre at Al-Kharj in Saudi Arabia, while one Majaheem and one Sofor were sampled from Alwatani camel dairy farm in Saudi Arabia. The Omani samples were from the King Fahad camel herd (**Table 1**).

**Table 1:** Sampling summary for the different Sudanese and Arabian Peninsula camel populations included in this study.

Population	Number of samples	Type	Location	Region	Type of data^1^	Type of analysis
Bishari	7	Racing camel	Kassala State	Eastern Sudan	GBS	Genetic diversity and selection analyses
Anafi	8	Racing camel	Kassala State	Eastern Sudan	GBS	Genetic diversity and selection analyses
Lahawi	10	Packing camel	Kassala State	Eastern Sudan	GBS	Genetic diversity and selection analyses
Kenani	10	Packing camel	Elgazira State	Central Sudan	GBS	Genetic diversity and selection analyses
Arabif	9	Packing camel	Darfur State	Western Sudan	GBS	Genetic diversity and selection analyses
Majaheem	2	Packing camel	the Conservation and Genetic Improvement Center at Al-Kharj	Saudi Arabia	WGS	Genetic diversity analyses
Majaheem	1	Packing camel	Alwatania camel dairy farm	Saudi Arabia	WGS	Genetic diversity analyses
Sofor	1	Packing camel	Alwatania camel dairy farm	Saudi Arabia	WGS	Genetic diversity analyses
Omani	2	Racing camel	King Fahad’s camel herd	Saudi Arabia	WGS	Genetic diversity analyses

^1^GBS, genotyping-by-sequencing; WGS, whole-genome sequencing.

### Genotyping-by-Sequencing (GBS) Data

Ten milliliters of whole blood were collected from each Sudanese sample using EDTA VACUETTE^®^ tubes. Genomic DNA was extracted from these whole-blood samples using the DNeasy Blood and Tissue Kit (Qiagen) according to the manufacturer’s protocol. The quality and quantity of the DNA were evaluated using a NanoDrop Spectrophotometer (NanoDrop Technologies, USA) and by gel electrophoresis. The extracted DNA samples were genotyped using pair-end GBS technology incorporating two restriction enzymes, *MseI* and *EcoRI*, sequenced on the Illumina HiSeq 2000 platform by Novogene (Novogene Co., Ltd., Tianjin, China). Novogene trimmed adapters from the sequence reads and discarded raw read pairs if (1) they were contaminated with adapter sequences, (2) uncertain nucleotides constituted more than 10% of either read, or (3) if low-quality nucleotides (base Q_phred_ ≤ 5) constituted more than 50% of either read.

### Whole-Genome Sequence (WGS) Data

Genomic DNA of the six Arabian Peninsula dromedary samples was extracted from 5 ml whole blood from each sample using the DNeasy^®^ Blood and Tissue Kit (Qiagen) according to the manufacturer’s protocol. The extracted genomic DNA was sequenced using pair-end libraries on the Illumina HiSeq 2000 platform (Beijing Genomics Institute (BGI), China). BGI trimmed adapters from the sequence reads and discarded raw read pairs if low-quality nucleotides (base Q_phred_ ≤ 5) constituted more than 50% of either read.

### Processing of Sequence Data

The clean raw sequence reads of both the GBS and WGS data were mapped to the Arabian dromedary camel reference genome assembly (GCF_000767585.1) (Wu et al., 2014) using the *BWA-MEM* algorithm of Burrows–Wheeler Aligner (BWA) version 0.7.5a (Li and Durbin, 2010). This aligner employs local alignment which results in the ends of reads being soft-clipped if they fail to map sufficiently well. Picard tools version 1.119 (http://broadinstitute.github.io/picard/index.html) was used to sort the reads by coordinate and mark PCR duplicates. Marking duplicate reads results in those reads being ignored in downstream analyses both by the Genome Analysis Toolkit GATK version 4.1 (Mckenna et al., 2010) and SAMtools version 1.3.1 (Li, 2011). Mapped reads with insertions or deletions (indels) were realigned using GATK’s IndelRealigner.

Single-nucleotide polymorphisms (SNPs) were called across the WGS (Arabian Peninsula) and GBS (Sudan) data, separately, using GATK’s HaplotypeCaller and SAMtools mpileup. HaplotypeCaller employs a number of default read filters; these include: removing reads that fail platform/vendor checks, unmapped reads, secondary alignments, reads that are not well-formed, reads that fail the minimum mapping quality (20), reads marked as duplicates, and reads with a bad CIGAR string (for further details, refer to the GATK documentation available at https://software.broadinstitute.org/gatk). Variants identified by SAMtools mpileup were required to have a minimum read mapping quality of 20 (MAPQ20) and a minimum base quality of 20. For each dataset, the genotypes of SNPs that were identified by both variant detection algorithms were retrieved from SAMtools, resulting in a total of 1,065,798 SNPs in Arabian Peninsula samples and 402,077 SNPs in the Sudanese populations. These variants were further hard-filtered using the VariantFiltration tool of GATK. This included removing variants with low quality by depth (QD < 2) to normalize variant quality and avoid inflation in the presence of deep coverage, removing variants indicating a high probability of strand bias (FS > 60), removing variants with a low root mean score mapping quality (MQ < 40), removing variants with low variant site quality (QUAL < 30), retaining variants where the mapping qualities of reads supporting the reference and alternate allele did not exhibit a bias for either allele (MQRankSum < −12.5), and retaining variants where their positions did not exhibit a bias toward the ends of reads (ReadPosRankSum < −8). The remaining variants were subsequently filtered using bcftools version 1.6 (Li, 2011) to retain those with a depth of coverage (DP) ≥ 5, and with a per-SNP DP within three standard deviations (SD) of the mean DP across all samples for a given dataset. The transition/transversion (Ts/Tv) ratio was calculated before and after filtering using bcftools stats. Variants on the mtDNA (scaffold NC_009849.1), indels, and SNPs that were not bi-allelic were also removed. A total of 206,415 and 1,028,936 SNPs were retained for the GBS and WGS data, respectively (**Supplementary Table S1**).

The filtered genotype data from the WGS and GBS datasets were merged, retaining a total of 1,173,266 SNPs common to both, and is herein referred to as the merged dataset. The filtered genotype data from the GBS dataset, independent of the WGS dataset, is herein referred to as the Sudanese dataset. A quality control (QC) steps was performed using the *check.marker* function implemented in the GenABEL package (Aulchenko et al., 2007) for R software version 2.15.1 (R Development Core Team, 2012). SNPs with minor allele frequency (MAF) less than 5% and call rate less than 95% were excluded from each dataset. A breakdown of the SNPs failing QC in each dataset is provided in **Table 2**. The final numbers of SNPs after QC were 12,920 and 39,843 in the merged and Sudanese datasets, respectively. For the genetic diversity analyses, SNPs with high linkage disequilibrium (LD) (*r^2^* > 0.1) were filtered out using the *indep-pairwise* tool (–indep-pairwise 50 10 0.1) in PLINK version 1.9 (Purcell et al., 2007). A total of 7,273 and 26,804 SNPs were removed from the merged and Sudanese datasets, respectively. For the pooled heterozygosity (*Hp*) analyses of the Sudanese dataset, the MAF criteria were not applied as the statistic is specifically testing for large deviations in heterozygosity levels and as such is sensitive to MAF. The LD filter was also excluded, as regions under selection are expected to accommodate SNPs in LD, and so removing these reduces the power of the analysis. The QC filtering was applied to the combined Sudanese, packing, and racing camel subsets independently, resulting in 49,943 SNPs in the combined Sudanese, 48,808 SNPs in packing and 46,535 SNPs in racing camels. None of the samples from either QC-filtered dataset (merged and Sudanese) exhibited a genotyping call rate <95%, or an identity by state (IBS) ≥ 90% with any other sample.

**Table 2:** Summary of SNPs numbers following quality control (QC) process.

	Datasets	
	Merged dataset	Sudanese dataset
	**Raw SNP number**	
	1,173,266	206,415
Quality control criteria		
MAF^1^ < 5%	105,394	39,937
Call rate < 95%	1,158,312	156,472
Both MAF and call rate	103,360	29,837
Linkage disequilibrium (LD)^2^		26,804
	**Final SNP number**	
Before LD pruning	12,920	39,843
After LD pruning		13,039

^1^Minor allele frequency.

^2^For genetic diversity analyses.

### Genetic Diversity Analyses

The mean heterozygosity and the inbreeding coefficient (*Fis*) of the different camel populations were computed from the merged dataset using the *hom* function implemented in GenABEL (Aulchenko et al., 2007). The two-sample Mann–Whitney U test was used to test for statistically significant differences in the heterozygosity and *Fis* values between the Arabian Peninsula and Sudanese camels, and between the different Sudanese populations. The one-sample Mann–Whitney U test was used to check if the *Fis* values of each of the distinct Sudanese populations, the combined Sudanese populations, and the Arabian Peninsula camels were significantly different from zero. Principle component analyses (PCA) were conducted in R using the *prcomp* function on both datasets to determine the genomic relationship between Arabian Peninsula and Sudanese camels, and separately among the Sudanese camels only.

To assess levels of genetic ADMIXTURE, analyses were performed using ADMIXTURE 1.23 (Alexander et al., 2009) on each dataset, assuming a number of clusters ranging from 1 to the number of populations sampled in each dataset (merged = 8; Sudanese = 5). In each case, 200 bootstrap iterations were performed. The optimal number of clusters was determined following Evanno et al. (2005) by calculating the second order rate of change of the likelihood for each *K* value. Figures of the ancestry assignments were plotted using the *ggplot2* package (Wickham, 2009) for R.

The relatedness of samples was evaluated in PLINK using the –make-rel tool. This tool estimates genetic relatedness among genome-wide SNPs based on the unadjusted *A_jk_* statistic described in Yang et al. (2011), which ranges from 0 for an unrelated pair of individuals to 1 when comparing an individual to itself. Yang et al. (2011) consider a pair of individuals to be related where *A_jk_* > 0.025. The relationship matrix was plotted using the ggplot2 package for R.

### Signatures of Selection Analyses

#### Pooled Heterozygosity (Hp) Analysis

*Hp* values were calculated using the formula described by Rubin et al. (2010): *Hp* = 2 ∑ n_MAJ_ ∑ n_MIN_/(∑ n_MAJ_ + ∑ n_MIN_)^2^. The analysis was performed in 50-kb sliding windows with a 25-kb step on all of the Sudanese camels, and independently for the Sudanese packing and racing types. After discarding windows supported by only a single SNP, the major and minor allele counts for each SNP were recorded (n_MAJ_ and n_MIN_, respectively), and *Hp* values of each 50-kb window were calculated, which were subsequently Z-transformed: ZHp = *Hp* – median *Hp*/SD *Hp*. Values in the extreme lower 0.1% tail of the empirical distribution of each analysis were considered significant (all Sudanese Zhp = −2.51; packing Zhp = −2.45; racing Zhp = −2.22) (**Supplementary Figure S2**). Windows with significant Zhp values that overlapped were merged into single regions.

#### Fixation Index (*Fst*) Analysis

Fixation index (*Fst*) analysis (Weir and Cockerham, 1984) was calculated between Sudanese packing and racing camels in 50-kb windows with a 25-kb step using VCFtools version 0.1.13 (Danecek et al., 2011). As with the *Hp* analyses, windows comprising only a single SNP were discarded. Values in the extreme upper 0.1% tail of the *Fst* distribution were considered significant (*Fst* = 0.206) (**Supplementary Figure S2**). Windows with significant *Fst* values that overlapped were merged into single regions.

### Functional Characterization of Candidate Regions of Selection

The positions of the significant windows from each analyses were cross-referenced against the genes annotated in the Arabian Peninsula dromedary camel reference genome assembly using the *intersectBed* function from the *BedTools* software (Quinlan and Hall, 2010). From each analysis, a background gene set was also identified by retrieving all genes that intersect with all windows tested. Gene-set over-representation analyses were performed using the ConsensusPathDB-human (CPDB) online tool (http://cpdb.molgen.mpg.de/CPDB). From each analysis, the significant and background gene lists were analyzed, and the significantly enriched biological pathways and gene ontology (GO) terms were recorded. Pathways and GO terms that remained significant after false discovery rate (FDR) correction (*q*-value < 0.05) were considered for discussion. A review of the literature for the genes identified from each analysis was also conducted to evaluate their relevance to adaptation to the desert environment, production, and performance traits.

## Results

### Genetic Diversity and Relationship Among the Camel Populations

The mean observed heterozygosity in Arabian Peninsula (0.560 ± 0.003) was significantly higher than within Sudanese camel populations (0.347 ± 0.003) (*P*-value < 0.0001). Among Sudanese camels, heterozygosity values were not significantly different (*P*-values > 0.05) ranging from 0.341 in Anafi to 0.353 in Bishari (**Table 3**). The mean *Fis* value of Arabian Peninsula samples (−0.814 ± 0.01) was significantly lower than in Sudanese camel populations (−0.123 ± 0.033) (*P*-value < 0.0001). As with the heterozygosity values, the *Fis* values among the Sudanese camel populations were not significantly different from each other (*P*-value > 0.05) (**Table 3**). *Fis* values of the Arabian Peninsula and Sudanese dromedary populations were all significantly different from zero (*P*-value < 0.05) (**Supplementary Table S2**). The camels sampled from Arabian Peninsula exhibited higher relatedness (median *A_jk_* = 0.347 ± 0.093) than those sampled from Sudan (median *A_jk_* = −0.007 ± 0.018) (**Supplementary Figure S3**, **Supplementary Figure S4** and **Supplementary Table S3**).

**Table 3:** Summary of observed heterozygosity and inbreeding coefficient (*Fis*) for each camel population.

Population	Mean observed heterozygosity	Heterozygosity standard deviation	Mean Fis	Fis standard deviation
Majaheem + Sofor (Alwatania camel dairy farm)	0.56	0.003	−0.811	0.01
Majaheem (the conservation and genetic improvement center)	0.562	0.002	−0.820	0.007
Omani	0.559	0.005	−0.810	0.016
Arabi	0.348	0.009	−0.126	0.029
Kenani	0.345	0.008	−0.118	0.026
Lahawi	0.348	0.009	−0.127	0.029
Anafi	0.341	0.010	−0.105	0.032
Bishari	0.353	0.015	−0.141	0.050
All Arabian Peninsula	0.560	0.003	−0.814	0.011
All Sudanese	0.347	0.010	−0.123	0.033

**Figure 1:**
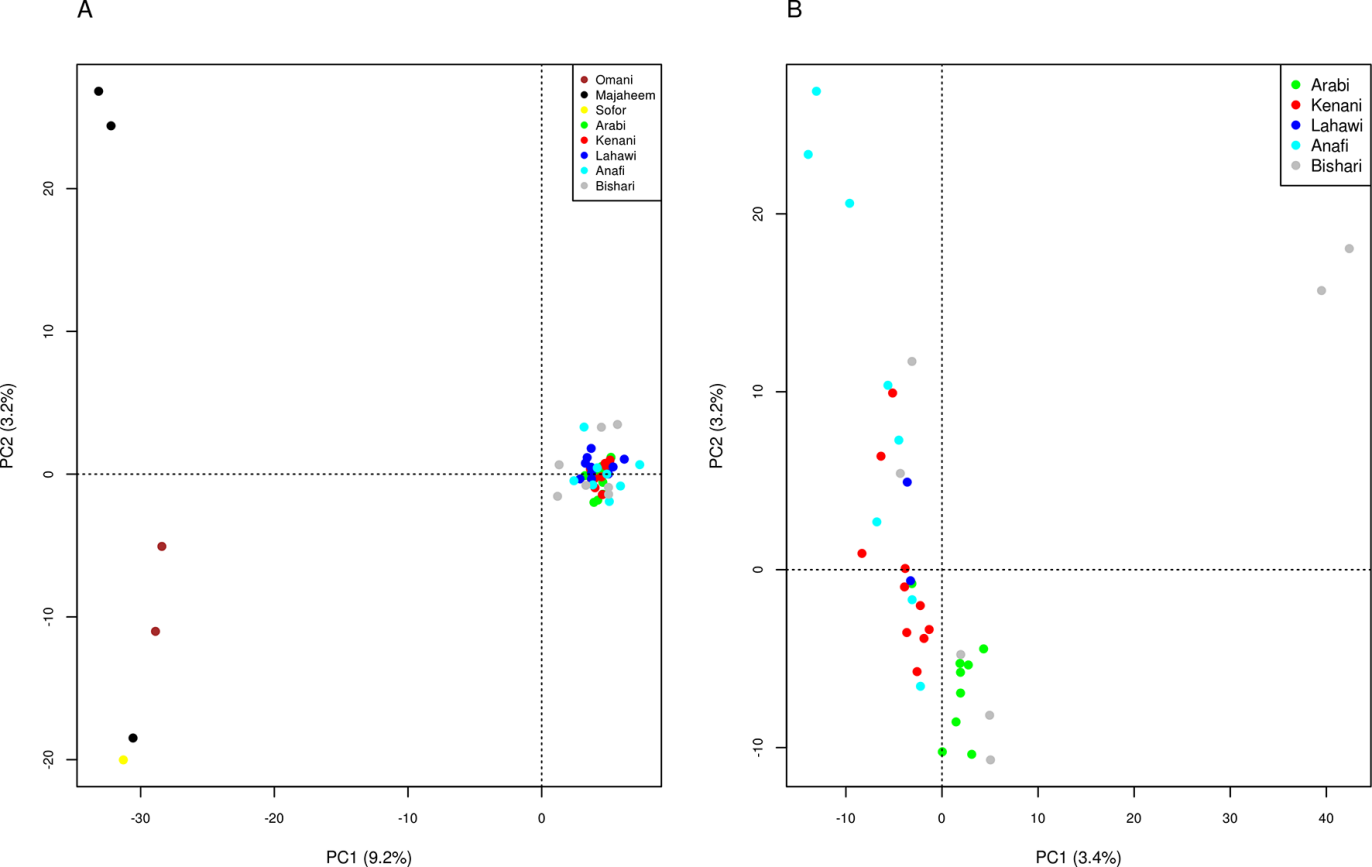
Principle components analysis, components 1 and 2 for **(A)** camel populations from Sudan and the Arabian Peninsula and **(B)** camel populations from Sudan only.

Principal component analysis (PCA) of the merged dataset differentiates the camel populations of Sudan from the Arabian Peninsula along the first principal component (PC1), which explains 9.2% of the total variation. PC2, which accounts for 3.2% of the total variation, distinguishes the two Majaheem from Al-Kharj from the other Arabian Peninsula dromedary (**Figure 1A**), while PC3, which explains 2.8% of the total variation, separates the Omani camels from the other Arabian Peninsula dromedary (**Supplementary Figure S5**). Analysis of the Sudanese camels alone reveals PC1 and PC2 to explain only 3.4 and 3.2% of the total variation, respectively. This indicates that the populations sampled are relatively homogeneous. Nonetheless, the variance of data along these components indicates two Bishari and three Anafi to diverge from the origin for PC1 and PC2, respectively (**Figure 1B**).

**Figure 2:**
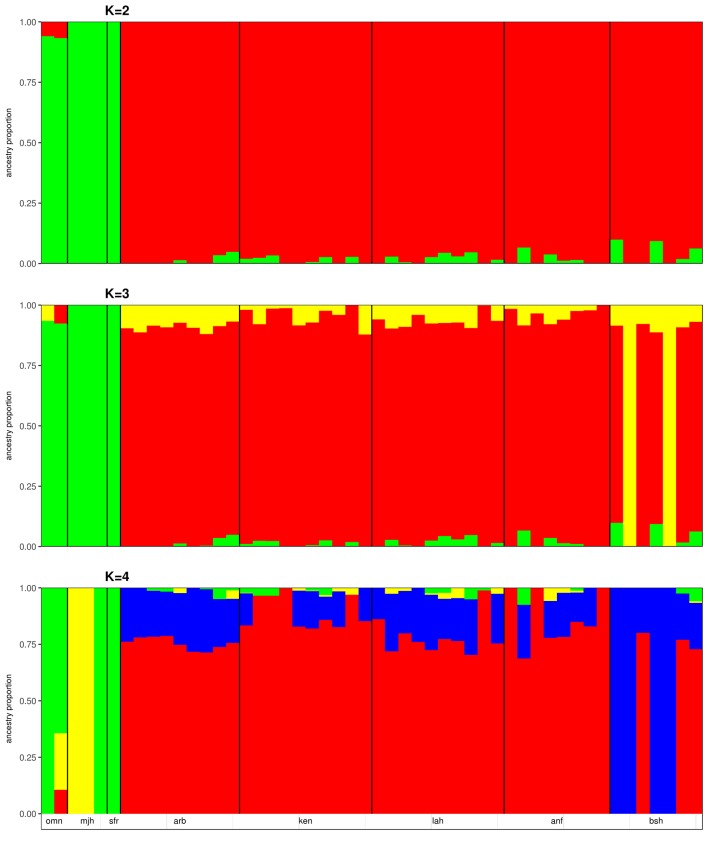
ADMIXTURE plots of Sudanese and Arabian Peninsula camels for cluster values (*K*) from 2 to 4. omn, Omani; mjh, Majaheem; sfr, Sofor; arb, Arabi; ken, Kenani; lah, Lahawi; anf, Anafi; bsh, Bishari.

We applied the *ΔK* approach of Evanno et al. (2005) to identify the optimal number of genetic backgrounds from the results of the ADMIXTURE analyses of the merged dataset and the Sudanese dataset. In both cases, the optimal number of genetic backgrounds is found to be two (**Supplementary Figure S6**). This is not surprising in the merged dataset, given the differentiation of samples collected from Sudan and the Arabian Peninsula by PCA (**Figure 1A**). At *K* = 3, a degree of ADMIXTURE is observed among the Sudanese camels, with two samples from Bishari providing the signal for the 3^rd^ genetic background (**Figure 2**). This pattern of ADMIXTURE is broadly observed at *K* = 2 in the independent analysis of Sudanese camels (Sudanese dataset; **Supplementary Figure S7**), although the extent of ADMIXTURE is slightly larger. At *K* < 4, the camels sampled from the Arabian Peninsula exhibit a more homogenous genetic background, distinct from the Sudanese camels with traces of Sudanese ancestry found in Omani camels (**Figure 2**). This distinction can also be observed at *K* values from 5 to 7 (**Supplementary Figure S8**). At *K* = 4 and 8, the two Majaheem samples from the Conservation and Genetic Improvement Centre provide a separate genetic background, while a single Majaheem camel retains a background shared with the Sofor sample which is also the dominant background in the Omani camels (**Figure 2** and **Supplementary Figure S8**).

### Candidate Signatures of Positive Selection

The mean *Hp* value across all sliding windows analyzed for the three Sudanese camel subsets (all Sudanese, packing, racing) was 0.24 ± 0.09. The analysis of all Sudanese camels returned 267 significant windows representing 176 regions. The subsets of packing and racing camels returned 281 and 472 significant windows representing 189 and 308 regions, respectively (**Table 4** and **Supplementary Table S4**), of which 133 were common to both camel types (**Supplementary Table S5**). A total of 159 and 148 regions identified in packing and racing camels, respectively, overlapped with the significant regions identified across all Sudanese camels. In total, 132 regions were found to overlap across each analysis (**Supplementary Table S5**).

**Table 4:** Summary of windows, regions, and genes identified in signatures of selection analyses in Sudanese camel.

	Signatures of selection analysis			
	*Hp*			*Fst*
	All Sudanese camels	Packing camels	Racing camels	
Number of windows	267	281	472	13
Number of merged regions	176	189	308	8
Number of genes	132	137	255	11
Number of regions with genes	86	99	161	5

The *Fst* analysis revealed 13 out of 13,097 windows to exhibit high genetic differentiation (*Fst* > 0.206) between racing and packing camels from Sudan. These windows represented eight distinct regions (**Table 4** and **Supplementary Table S6**).

### Functional Characterization of Candidate Regions of Selection

The *Hp* analysis identified 132, 137, and 255 genes to intersect with 86, 99, and 161 regions in all Sudanese, packing, and racing camels, respectively (**Table 4** and **Supplementary Table S7**). While the *Fst* analysis identified 11 genes to overlap with five genetically differentiated regions between Sudanese packing and racing camels (**Table 4** and **Supplementary Table S8**). These genes are associated with different biological pathways, including immune response, fertility, milk content, energy homeostasis, and chondrogenesis (**Table 5**). No biological pathways were found to be significantly over-represented in the genes identified from the analyses of all Sudanese and packing camels. While a single GO term (monocarboxylic acid transmembrane transporter activity) was found to be significantly enriched in the analysis of all Sudanese camels. Moreover, a single biological pathway associated with liver kinase B1 (*LKB-1*) signaling was found to be significantly over-represented in Sudanese racing camels (**Table 6** and **Supplementary Table S9**). In total, four biological pathways and five GO terms are significantly enriched among the genes identified from the *Fst* analysis between Sudanese racing and packing camels (**Table 6** and **Supplementary Table S9**).

**Table 5:** Genes intersecting the pooled heterozygosity (*Hp*) and genetic differentiation (*Fst*) candidate regions.

Functional category	Gene ID	Gene description	Type of candidate region
Immune response	C9	Complement component C9	Packing/racing/all Sudan camels (Hp region)
	IL6R	Interleukin-6 receptor subunit alpha	Packing/racing/all Sudan camels (Hp region)
	CCR8	C–C chemokine receptor type 8	Packing/all Sudan camels (Hp region)
	CX3CR1	CX3C chemokine receptor 1	Packing/racing/all Sudan camels (Hp region)
	LOC105100014	Complement receptor type 1-like	Racing camels/all Sudan camels (Hp region)
	C1QTNF8	Complement C1q tumor necrosis factor-related protein 8	Packing/all Sudan camels (Hp region)
Fertility	LOC105094930	Olfactory receptor 1S1-like	Racing camels (Hp region)
	LOC105094932	Olfactory receptor 5B12	Racing camels (Hp region)
	LOC105094933	Olfactory receptor 5B3-like	Racing camels (Hp region)
	ESR1	Estrogen receptor	Racing camels (Hp region)
	SPACA5	Sperm acrosome-associated protein 5	Packing/racing/all Sudan camels (Hp region)
Milk content	PICALM	Phosphatidylinositol-binding clathrin assembly protein	Packing/racing/all Sudan camels (Hp region)
Chondrogenesis	LOC105087163	Chondroitin sulfate proteoglycan 4-like	Packing/racing/all Sudan camels (Hp region)
	CRLF1	Cytokine receptor-like factor 1	Fst region
	CHSY1	Chondroitin sulfate synthase 1	Racing (Hp region)
Energy homeostasis	ESRRG	Estrogen-related receptor gamma	Packing/racing/all Sudan camels (Hp region)
	CRTC1	CREB-regulated transcription coactivator 1	Fst region
Running performance	NAA16	N (alpha)-acetyl transferase 16	Fst region

**Table 6:** Summary of significantly enriched biological pathways and gene ontology (GO) terms following gene-set over-representation analyses.

	Signatures of selection analysis						
	*Hp*					*Fst*	
	All Sudanese camels	*q*-value^1^	Packing camels	Racing camels	*q*-value^1^		*q*-value^1^
Biological pathway	None	None	None	LKB1 signaling events	0.015	Brain-derived neurotrophic factor (BDNF) signaling pathway	0.006
						Human T-cell leukemia virus 1 infection—Homo sapiens (human)	0.006
						Signaling by interleukins	0.006
						Cytokine signaling in immune system	0.011
Gene ontology (GO) term	Monocarboxylic acid transmembrane transporter activity	0.019	None	None		Ribosome binding	0.001
						Ureteric bud development	0.032
						Mesonephros development	0.032
						Kidney epithelium development	0.032
						Ribonucleoprotein complex binding	0.007

^1^The q-values in all analyses are derived from false discovery rate correction.

## Discussion

In this study, we investigated the genetic diversity and relationship between camels sampled from Sudan and the Arabian Peninsula using genotype data derived from GBS and WGS. The migration of camels to Sudan from their putative center of domestication in the Arabian Peninsula may have resulted in a founder effect coupled with genetic drift that can explain the reduced heterozygosity observed in the Sudanese camels we studied. Populations that are closer to their centers of domestication are expected to exhibit higher levels of heterozygosity than more distant populations. Thus, the higher level of heterozygosity observed in the camels sampled from the Arabian Peninsula compared to those from Sudan offers some support for the southeast of the Arabian Peninsula as a center of domestication. Investigating a greater breadth and depth of sampling throughout Africa and the Arabian Peninsula will provide further context for these interpretations.

The Arabian Peninsula and Sudanese camel populations both exhibit mean negative *Fis* values significantly different from zero, which may be suggestive of low levels of inbreeding among these populations as a consequence of their historical use in transportation and trading—which might be associated with continuous interbreeding and gene flow. We sought to evaluate the relatedness of samples using the *A_jk_* statistic, which indicated the majority of Sudanese camels to be unrelated, whereas all of the camels from the Arabian Peninsula returned high values (*A_jk_* > 0.025). These high values observed across the Arabian Peninsula camels are likely an artifact of the small sample size that can result in severely biased estimates (Wang, 2017).

PCA and ADMIXTURE analyses revealed a phylogeographic distinction between the Arabian Peninsula and Sudanese camels. This might be attributed to the geographical isolation of these two populations by the Red Sea, hindering gene flow between them. Such genetic distinction has been observed between camel populations from Kenya, the Arabian Peninsula, and Pakistan (Mburu et al., 2003). We also observed traces of Sudanese ancestry in Omani camels, which might possibly result from historical interbreeding while following the trading routes connecting Africa with the south of Arabian Peninsula (Andree-Salvini, 2010). These results imply that the history of dromedary camel migration and interbreeding is highly complex and requires further investigation. A recent study by Almathen et al. (2016) showed that Sudanese camels are genetically distinct to camels from the south of the Arabian Peninsula but not the north. The discrepancy between that study and our results is likely to be due to our limited sampling of the Arabian Peninsula, which did not adequately capture the broader diversity of camel populations present. Furthermore, Almathen et al. (2016) employed autosomal microsatellite markers, whereas our genotypic data was derived from next-generation sequencing and is potentially more informative.

We observed no clear genetic structuring of the different Sudanese camel populations studied, providing further support of continuous gene flow. The two Bishari and three Anafi divergent camels exhibited marginally higher relatedness with one another than with other camels sampled from the same regions (**Supplementary Figure S3**). This likely accounts for their divergence in the PCA (**Figure 1B**). The lack of genetic distinction between the packing and the racing camels reflects a common practice in Sudan of breeding racing camels from East Sudan with packing camels in the West to improve the performance of packing camels. This is also reflected in the mean negative *Fis* values in the Sudanese dromedary camel populations analyzed.

The camels from the Arabian Peninsula were broadly found to be homogeneous in the ADMIXTURE analyses. PCA differentiated Omani samples from the rest of the Arabian Peninsula along the 3^rd^ principal component. The 2^nd^ principle component, although explaining little variation, differentiated the Majaheem and Sofor samples from the Alwatania camel dairy farm from the two Majaheem samples from the conservation and genetic improvement center. This was also observed in the ADMIXTURE analysis at K = 4 and K = 8. This suggests that camels from the Arabian Peninsula might exhibit a degree of genetic structure based on their breeding location and/or geographical origin. This observation however, which agrees with the findings of Almathen et al. (2016), requires further investigation using a larger and more diverse sample of camels from the Arabian Peninsula.

We further investigated the Sudanese camels for signatures of positive selection. A number of genomic regions exhibiting reduced heterozygosity were identified across the Sudanese camels, and within the subsets of packing and racing camels (**Table 4**). The *Fst* analysis, however, revealed few genetically differentiated regions between racing and packing camels. There was no overlap between the regions identified by the *Fst* analysis and those identified by the *Hp* analysis. This is not entirely unexpected as the two analyses are better-suited to different selection time frames, whereby *Fst* focuses on more recent selection than the *Hp* statistic (Oleksyk et al., 2010).

The regions identified from these analyses host a number of genes, for which gene set over-representation analyses and extensive literature review revealed a number to be of functional interest. In particular, we identified genes linked to the immune response, fertility, milk content, chondrogenesis, energy homeostasis, and running performance (**Table 5**). Whether or not these genes are linked to the domestication of dromedary camels from their wild ancestors, as found in sheep and goats (Alberto et al., 2018), requires further validation using genomic data from the wild ancestor of dromedary camels.

Examples of genes involved in the immune response include members of the complement system (*C9, LOC105100014*, and *C1QTNF8*), CX3C chemokine receptor 1 (*CX3CR1*), and interleukin-6 receptor subunit alpha (*IL6R*) genes, both of which were identified in packing and racing camels. The complement system links the innate and adaptive immune responses, mediating responses to inflammatory triggers through a co-ordinated enzyme cascade (Nesargikar et al., 2012). *CX3CR1* and interleukin-6 (*IL-6*) are both involved in inducing inflammation in response to infection and tissue injuries (Ishida et al., 2008; Tanaka et al., 2014), while *IL-6* is also involved in native T-cell differentiation (Tanaka et al., 2014). The chemokine receptor type 8 (*CCR8*) gene, identified in packing camels, plays a role in regulating the immune system by controlling regulatory T-cell activity (Coghill et al., 2013). Genes in this category have also been identified to be under adaptive evolution in dromedary and Bactrian camels in a study by Wu et al. (2014).

Genes involved in fertility have been identified in both packing and racing Sudanese dromedary camels. These include the *ESR1* gene which encodes the estrogen receptor alpha that mediates estrogen action on target tissues. Mice whose estrogen receptor-alpha gene has been knocked out demonstrate complete infertility (Matthews and Gustafsson, 2003) and impaired spermatogenesis (Couse et al., 2001). Olfactory receptors have been shown to be expressed in mature sperm (Parmentier et al., 1992; Vanderhaeghen et al., 1993) and to play a role in oocyte fertilization upon interaction with chemo-attractants secreted from oocyte-cumulus cells (Spehr et al., 2003; Fukuda et al., 2004). The sperm acrosome-associated protein 5 gene (*SPACA5*) plays a role in acrosome reaction and the fertilization process (Agarwal et al., 2015). Fertility-associated genes have also been found to be under selection in indigenous African zebu cattle (Bahbahani et al., 2018a; Bahbahani et al., 2018b) in order to maintain fertility in the harsh environment (Skinner and Louw, 1966; Hansen, 2004).

An example of a gene likely to be a feature of adaptation to the desert environment is *ESRRG*. This gene, which is in candidate regions identified in both camel types, is related to energy homeostasis. *ESRRG* encodes the estrogen-related receptor gamma protein, which is involved in regulating metabolism and energy production in cells (Eichner and Giguere, 2011). A transition polymorphism (rs1890552 A > G) in this gene has been found to be associated with decreased levels of fasting glucose in humans (Kim et al., 2017b). The gene-set over-representation analysis in racing camel also identified the over-representation of genes associated with *LKB1* signaling pathway, which is associated with regulating cellular energy metabolism in eukaryotic cells (Alessi et al., 2006). Genes in this category have also been found to be under adaptive evolution in dromedary and Bactrian camels (Wu et al., 2014).

A gene which encodes the phosphatidylinositol-binding clathrin assembly protein (*PICALM*), which is involved in regulating milk content, was identified in both racing and packing camel types. Variants in *PICALM* have been associated with α_S1_-casein content in cattle milk (Sanchez et al., 2017). This gene is therefore of potential future interest to improve the productivity of dromedary camels through genomic selection breeding programs (Al Abri and Faye, 2019).

A number of genes identified in candidate regions in packing and racing camels were found to be associated with chondrogenesis. These include the *LOC105087163* gene which encodes the chondroitin sulfate proteoglycan 4 protein. Members of this protein family, such as aggrecan and versican, have been found to be involved in cartilage and limb joint formation (Watanabe et al., 1994; Choocheep et al., 2010). Another chondrogenesis-related gene identified, *CHSY1*, encodes the chondroitin sulfate synthase 1 protein, which, when knocked-out in mice, results in cartilage impairment, aberrant joint formation, and decreased bone density (Wilson et al., 2012). The identification of genes associated with chondrogenesis in packing dromedary camels might be a reflection of the historical use of camels in trading and transportation, which likely placed chronic physical demands on these animals similar to other livestock employed in the provision of draught power. The signatures of selection identified for genes associated with dairy traits in racing camels might also be a historical reflection of the general use of camels by nomads in the provision of milk.

A number of genes associated with energy homeostasis, chondrogenesis, and running performance were identified within the genetically differentiated regions between racing and packing camels. One such example is CREB-regulated transcription coactivator 1 (*CRTC1*). This gene is linked to energy homeostasis, which is likely to be an important function in racing camels in order to maintain their stamina during racing competitions. Studies in mice support this, indicating that the gene is associated with energy balance (Altarejos et al., 2008). Another candidate gene identified was cytokine receptor-like factor 1 (*CRLF1*), which plays a role in chondrogenesis. The expression of this gene has been shown to be up-regulated in mice chondrocytes upon stimulation by TGF-beta factor which, together with its co-factor cardiotrophin-like cytokine factor 1 (CLCF1), induces proliferation of chondrocyte precursors (Stefanovic and Stefanovic, 2012). A final example, associated with running performance, is the N(alpha)-acetyl transferase 16 (*NAA16*) gene, which has been correlated with average running speed in mice (Kelly et al., 2014). Interestingly, three out of the five significantly enriched gene ontology terms in the *Fst* analysis genes are associated urinary tract and kidney development, which might be linked with the running performance of racing camels (**Table 6**). A study by Leikis et al. (2006) has revealed reduced exercise performance in patients with chronic kidney disease at stages 3 and 4. This reduction in exercise capacity, and associated muscle strength, was also been linked with renal function failure. Moreover, the impairment in muscle K^+^ level regulation, which is associated with renal function failure (Bergstrom et al., 1983), has also been found to be linked with muscle fatigue (Sejersted and Sjogaard, 2000). This requires further investigations through detailed physiological studies on racing and packing dromedary camels.

To further our understanding of the genetic mechanisms underlying phenotypic traits of importance in dromedary camel, a number of actions are required. In the first instance, the reference genome is currently at the scaffold stage and would greatly benefit from the application of long-read single-molecule sequencing technologies coupled with optical mapping to improve its quality (Mostovoy et al., 2016; Weissensteiner et al., 2017). Improving the assembly of the reference genome to reach the chromosome stage is essential to facilitate detailed analyses for signatures of selection. The recent construction of two whole-genome radiation hybrid panels for dromedary camels by Perelman et al. (2018) is a promising first step toward improving the assembly. Secondly, in parallel to work improving the contiguity of the reference genome is the need for functional analyses on the annotated genes. Improving our understanding of the function of genes provides greater context toward interpreting genotype–phenotype associations. Thirdly, the detailed characterization of camels using standard phenotypic and morphometric parameters is required to more accurately classify the different camel types. Using WGS data in future studies instead of GBS will provide greater resolution to analyses, and less redundancy of data, while increasing the breadth and depth of sampling will enable more reliable calculations of genetic diversity parameters.

## Conclusion

We have reported here for the first time the phylogeographic classification between the Arabian Peninsula and Sudanese dromedary camel populations using WGS and GBS data. We identified a number of genomic regions under positive selection hosting genes of putative relevance to Sudanese packing and racing camels, including genes potentially associated with dairy traits and running performance. The results of this study call for further investigation of the genome of dromedary camel using larger and more diverse populations to better identify variants associated with their important phenotypes. The results of which can support the development of informed breeding programs with the aim to improve the productivity and performance of dromedary camels.

## Data Availability

The Arabian Peninsula camel full genome sequence data and the genotyping-by-sequencing data of Sudanese camels are publicly available from the European Nucleotide Archive (ENA) with the Bioproject accession number PRJEB32117.

## Ethics Statement

Standard techniques were used to collect blood. The procedure was reviewed and approved by Faculty of Veterinary Science, University of Nyala, Sudan.

## Author Contributions

HB, HM and OH conceived and designed the project. ES collected the dromedary blood samples from Sudan. HM and FA contributed sequence data for the Sudanese and Arabian Peninsula samples, respectively. HB and DW performed bioinformatic analysis. HB, DW and OH wrote the manuscript. All authors have commented upon and agreed on the contents of the manuscript.

## Funding

This work is part of the project “Agricultural growth, capacity building for scientific preservation of livestock breeds in Sudan.” The project was supported by a grant from the Korea- Africa Economic Cooperation Trust Fund through the African Development Bank and co-funded by the livestock CGIAR-CRP. We would like to extend our gratitude to King Faisal University (Saudi Arabia) for their research award (Grant F289) to sequence the genomes of the six Arabian Peninsula dromedary camel samples.

## Conflict of Interest Statement

The authors declare that the research was conducted in the absence of any commercial or financial relationships that could be construed as a potential conflict of interest.

The handling editor and reviewer PO-TW declared their involvement as co-editors in the Research Topic.
