# Supplementary material for: Genome Diversity and Signatures of Selection for Production and Performance Traits in Dromedary Camels
Source: Front Genet. 2019 Sep 19;10:893. doi: 10.3389/fgene.2019.00893 (PMC6761857; doi:10.3389/fgene.2019.00893)
Supplement: Supplementary file 10 [file DataSheet_1.zip › Bahbahani_etal_SuppInfo_revised_version.docx]

Supplementary Material

**Genome diversity and signatures of selection for production and performance traits in dromedary camels**

Hussain Bahbahani^*^, Hassan H. Musa^*^, David Wragg, Eltahir S. Shuiep, Faisal Al mathen and Olivier Hanotte

**Correspondence:**

Hussain Bahbahani

h.bahbahani@hotmail.com

[hussain.bahbahani@ku.edu.kw](mailto:hussain.bahbahani@ku.edu.kw)

Hassan Musa

[hassanhm@uofk.edu](mailto:hassanhm@uofk.edu)

**Supplementary Figure 1**: The map of Sudan showing the geographical locations of the sampled Sudanese camel populations.

**Supplementary Figure 2:** The empirical distribution of (A) Zhp values in Sudanese packing camels, (B) Sudanese racing camels, (C) combined Sudanese camels, (D) Fst values between Sudanese packing and racing camels. The solid line represents the significant threshold at the bottom 0.1% tail of Zhp distribution and top 0.1% tail of Fst distribution. Number of significant windows in each distribution is stated in each figure.

**Supplementary Figure 3**: A relatedness matrix based on *A_jk_* statistic between the camel samples included in the study. Pairs of individuals with *A_jk_* > 0.025 are marked with an (x). Lahawi= Cam_A, Kenani= Cam_K, Bishari= Cam_B, Anafi= Cam_An, Arabi= Cam_A, Omani= BAZ_Y and SMAMA, Sofor= N3136, Majaheem from the conservation and genetic improvement centre= ANF172235 and ANF171631, Majaheem from Alwatania camel dairy farm= N3160.

**Supplementary Figure 4**: A density plot of the *A_jk_* statistic on the different camel pairs. Pairs of individuals with *A_jk_* > 0.025 are classified as unrelated.

**Supplementary Figure 5**: A plot of the first and third principle components on all the Sudanese and Arabian Peninsula camel populations included in the study.

A

| K | Delta K |
| --- | --- |
| 1 | 0 |
| 2 | 89190000000 |
| 3 | 35560000000 |
| 4 | 31280000000 |
| 5 | 28080000000 |
| 6 | 32530000000 |
| 7 | 27190000000 |
| 8 | 31240000000 |

B

| K | Delta K |
| --- | --- |
| 1 | 0 |
| 2 | 77070000000 |
| 3 | 70380000000 |
| 4 | 66420000000 |
| 5 | 65180000000 |

**Supplementary Figure 6**: The second order rate of change of the likelihood function with respect to the cluster *K* (delta K) proposed by Evanno *et al* (2005) for (A) Admixture analysis on the merged Arabian Peninsula and Sudanese camel populations dataset (B) Sudanese camel populations only dataset.

**Supplementary Figure 7:** ADMIXTURE bar plots of only Sudanese camel populations (Sudanese dataset) for cluster values (*K*) from 2 to 5. arb: Arabi, ken: Kenani, lah: Lahawi, anf: Anafi, bsh: Bishari.

**Supplementary Figure 8:** ADMIXTURE bar plots of the merged dataset including Sudanese and Arabian Peninsula camels for cluster values (*K*) from 5 to 8. omn : Omani, mjh: Majaheem, sfr: Sofor, arb: Arabi, ken: Kenani, lah: Lahawi, anf: Anafi, bsh: Bishari.

**Supplementary Table 1: is published separately - see the supplementary material files.**

**Supplementary Table 2: is published separately - see the supplementary material files.**

**Supplementary Table 3: is published separately - see the supplementary material files**

**Supplementary Table 4: is published separately - see the supplementary material files**

**Supplementary Table 5: is published separately - see the supplementary material files**

**Supplementary Table 6: is published separately - see the supplementary material files**

**Supplementary Table 7: is published separately - see the supplementary material files**

**Supplementary Table 8: is published separately - see the supplementary material files**

**Supplementary Table 9: is published separately - see the supplementary material files**
